# Anger and aggressiveness in obsessive–compulsive disorder (OCD) and the mediating role of responsibility, non-acceptance of emotions, and social desirability

**DOI:** 10.1007/s00406-020-01199-8

**Published:** 2020-11-05

**Authors:** Barbara Cludius, Anna K. Mannsfeld, Alexander F. Schmidt, Lena Jelinek

**Affiliations:** 1 Department of Psychiatry and Psychotherapy, University Medical Center Hamburg-Eppendorf, Hamburg, Germany; 2 Department of Clinical Psychology and Psychotherapy, Ludwig-Maximilians University, Leopoldstr. 13, 80802 Munich, Germany; 3 Department for Social and Legal Psychology, Johannes Gutenberg-University, Mainz, Germany

**Keywords:** Anger, Aggression, Mediation, Obsessive–compulsive disorder, Implicit-association test, Emotion regulation

## Abstract

According to psychodynamic and cognitive models of obsessive–compulsive disorder (OCD), anger and aggression play an important role in the development and maintenance of the disorder. (Sub-) clinical samples with OCD have reported higher anger and anger suppression. Patients with checking-related symptoms of OCD showed a less aggressive self-concept as assessed by an Implicit Association Test (IAT). This study assessed anger and aggressiveness self-concepts in OCD as well as possible mediators of the link between OCD and aggressiveness. A total of 48 patients with OCD and 45 healthy controls were included. Measures included the State-Trait Anger Expression Inventory-II and an aggressiveness self-concept IAT (Agg-IAT). An inflated sense of responsibility, non-acceptance of emotions, and social desirability were tested as mediators. As expected, patients with OCD reported higher trait anger and anger suppression compared to healthy controls. Contrary to hypotheses, the aggressiveness self-concept (Agg-IAT) did not differ between groups. The inflated sense of responsibility mediated the relationship between group and anger suppression. Non-acceptance of negative emotions mediated the relationship between group and trait anger, as well as anger suppression. However, comorbidities and medication may account for some effect in anger suppression. Elevated trait anger and anger suppression in OCD patients could be explained by dysfunctional beliefs or maladaptive emotion regulation strategies. Emotion regulation therapy might help to enhance awareness and acceptance of emotions and possibly improve treatment outcomes.

**Electronic supplementary material:**

The online version of this article (10.1007/s00406-020-01199-8) contains supplementary material, which is available to authorized users.

## Introduction

According to psychodynamic theories, anger and aggression (i.e., anger expression)^1^ are thought to play an important role in the development and maintenance of obsessive–compulsive disorder (OCD; for an overview see [1]). Freud [2] postulated that the development of OCD symptoms can be explained by an underlying conflict between love and hate towards a significant other. This leads to a strong suppression of feelings of hate or anger due to love. According to Freud, suppressed anger leads to hypermorality (“Übermoral”), which he defined as high moral standards and exaggerated feelings of guilt and responsibility, the latter two being regarded as crucial contributors to OCD. Cognitive theories of OCD also suggest a close relationship between the disorder and (suppressed) anger insofar as OCD symptoms are said to result from dysfunctional beliefs [3–6]. One of those dysfunctional beliefs is an inflated sense of responsibility, similar to Freud’s postulation [2], through which individuals feel excessively responsible for preventing potential harm caused by themselves and others [5]. According to Rachman [3], an inflated sense of responsibility is associated with suppression of anger. He assumes that difficulties to express anger are caused by the internal attribution of responsibility to prevent harm from others, which results in guilt rather than aggression. This model is partly supported by previous research, according to which individuals with OCD regard themselves as fully responsible for preventing harm [7]. Following this notion, learning to express anger would result in a reduction of OCD symptoms [3].

In line with both psychodynamic and cognitive theories, the majority of previous studies revealed increased anger in both subclinical and clinical samples of OCD according to self-report [8–12]. The only exception is a study which did not find increased anger scores in OCD when comparing patients with OCD to healthy controls [13]. The majority of the studies assessed not only trait anger, but also suppressed anger or aggression. A study by Whiteside and Abramowitz [8] included a sample of college students who reported subclinical symptoms of OCD (i.e., OCD diagnosis was not an inclusion criterion). They found that college students who scored higher in OCD symptoms reported more suppression of anger. Among the studies including clinical samples of OCD, Moscovitch et al. [11] found that patients with OCD reported higher trait anger as well as a higher tendency to suppress anger compared to healthy controls. Patients with OCD diagnosis also reported more suppressed anger compared to healthy and clinical controls [9, 10]. In another study, patients with checking compulsions reported higher trait anger and higher aggression, but not higher anger suppression compared to healthy controls [12].

In line with psychodynamic theories, dual-process models of cognition [14] and empirical data suggest that aggression is determined by both automatic (e.g., [15]) as well as controlled (e.g., [16]) precursors. As self-report measures primarily assess controlled-tendencies, they should be complemented by indirect measures tapping into more automatic dispositions. So far, only one study has used an established indirect measure to assess aggression, namely an aggressiveness self-concept Implicit Association Test (Agg-IAT) in patients with OCD [17]. The Agg-IAT assesses the association between aggressiveness and the self [15, 16]. Contrary to results of self-report measures, patients with OCD did not differ from healthy controls regarding their aggressiveness self-concept. Furthermore, a subgroup of patients with checking compulsions showed less aggressive self-concepts than healthy controls in the Agg-IAT [17]. Even though this study showed surprising results regarding aggression in OCD, the interpretation of those results is limited due to a lack of complementary self-report measures of (suppressed) anger and aggression. Therefore, the question remains as to why patients with OCD report higher trait anger and anger suppression on self-report measures, whereas they concurrently show a similar or even less aggressive self-concept on the Agg-IAT. As suggested by cognitive theories (see above), one possible explanation for increased trait anger and anger suppression could be related to an inflated sense of responsibility [18]. Patients with OCD feel responsible for the safety of themselves and others. As it is impossible to fully prevent danger, patients could become frustrated and angry, which would explain higher anger scores on self-report measures [7]. However, as patients blame themselves rather than others for their inability to reduce harm, anger should be suppressed rather than expressed [3]. Another explanation could be that patients and healthy controls differ in the extent to which they respond to self-report measures according to social norms. Patients with OCD, especially those with checking compulsions, are thought to have high moral standards and a strong urge to behave correctly [3]. Thus, healthy controls may underreport anger due to social desirability, whereas patients with OCD may try to be as honest as possible to meet their high moral standards [3]. This would lead patients with OCD to score higher on self-report measures on anger compared to healthy controls, even with similar implicit aggressiveness self-concepts that are less amenable to social desirability [15]. A third explanation could be that patients with OCD and healthy controls differ in their personal appraisals of anger. As anger runs counter to patients’ high moral standards, it might be appraised as especially frightening. As suggested by Bardeen, Stevens, Murdock, and Lovejoy [19], such an inordinate interpretation of emotions (i.e., anger is frightening) might reduce emotional understanding and may lead to a non-acceptance of emotional states. Even though some researchers equate non-acceptance of emotions and emotional suppression (such as anger suppression), these two are conceptualized as distinct dimensions [20, 21]. Whereas non-acceptance of emotions is a value judgement of emotions (i.e., negative emotions are “bad”), emotional suppression is the act of not revealing that emotion. Thus, non-acceptance of emotions is only linked to negative outcomes, whereas anger suppression might, in some contexts, even be linked to favorable outcomes [22].

The aim of the current study was to extend the previous studies using only self-report measures or only the Agg-IAT [17]: First, we included both a self-report measure (STAXI-2) and an indirect measure (Agg-IAT) to assess (suppressed) anger and an aggressiveness self-concept simultaneously. Second, we examined possible mediating variables for the elevated anger scores on self-report measures, including measures of an inflated sense of responsibility, non-acceptance of emotional responses, and social desirability. Finally, patients with OCD as well as healthy controls were included in the study. We formulated the following hypotheses: Compared to healthy controls, patients with OCD show (a) a more peaceful self-concept in the Agg-IAT, (b) higher trait anger on the STAXI-2, and (c) higher anger suppression on the STAXI-2. Similar analyses were planned for a subgroup of patients with checking-related symptoms of OCD. This was first, because Rachman [3] claimed higher anger particularly in patients with checking-related symptoms of OCD and second, some of the previous studies have indeed found higher anger scores in this subgroup [12, 17]. We further hypothesized that an inflated sense of responsibility positively mediates the association between group (OCD patients vs. healthy controls) and (a) the aggressiveness self-concept (Agg-IAT), (b) trait anger (STAXI-2), and (c) anger suppression (STAXI-2). Furthermore, we planned to conduct the same analyses with non-acceptance of emotional responses and social desirability as mediators. To complement analyses and to explore the role of other potential mediators (i.e., comorbidity and medication), exploratory analyses were conducted for subgroups of patients: First, for patients with vs. without a current comorbid diagnosis of major depressive disorder (MDD), as some of the previous studies on anger and anger suppression in OCD have shown that results were influenced by depression [11, 13]. Second, for patients with and without comorbid anxiety disorder. Third, for patients with and without antidepressant medication, as the serotonergic system has been linked to aggression and as antidepressants such as serotonin reuptake inhibitors (SSRIs) have been found to reduce aggression in patients with personality disorder [23].

## Methods

### Participants

The sample consisted of 48 patients with an OCD diagnosis and 45 healthy controls. Patient recruitment was conducted within a larger project [24]. Recruitment took place through the OCD and anxiety ward of the University Medical-Center Hamburg-Eppendorf, Germany. Healthy controls were recruited by word of mouth. Participants were excluded if they met any of the following exclusion criteria: age lower than 18 or higher than 68, history of schizophrenic or affective-psychotic disorder, any neurological disorder, current alcohol or substance dependence, brain damage or intellectual disability (i.e., IQ < 70). Beyond that, healthy controls were excluded if they reported a history of depression, OCD or any present mental disorder. See Table 1 for patients’ interview- and questionnaire data. The required sample size for the main analyses was calculated using G*Power [25]. Previous studies assessing anger in patients with OCD [11, 12] revealed effect sizes ranging between *d* = 0.5 and *d* = 0.6. We calculated the required sample size for a *t* test comparing two independent groups with an effect size of *d* = 0.52 to achieve a test with 80% power at an error rate of *α* = 0.05. The analysis revealed that a total of 47 participants for each group would be necessary to find an effect. The larger study was registered with the German Clinical Trials Register (DRKS-ID: DRKS00012531) and approved by the Ethics Committee of the German Society for Psychology (#LJ032017).

**Table 1:** Descriptive overview of demographics and dependent variables: mean (standard deviation)

	OCD patients (*n* = 48)	Healthy controls (*n* = 45)	Statistics
Demographic characteristics			
Age (years)	32.46 (10.63)	37.29 (14.28)	*U* = 886.00, *p* = 0.14
Education (years)	11.33 (1.58)	11.93 (1.57)	*U* = 853.00, *p* = 0.07
Sex (m/f)	24/24	20/25	*χ*^2^(1) = 0.29, *p* = 0.59
Verbal intelligence (WST, IQ)	102.50 (12.65)	106.24 (8.88)	*U* = 859.50, *p* = 0.09
Social desirability (SES-17)	8.90 (2.09)^1^	9.24 (1.96)	*U* = 620.50, *p* = 0.41
Psychopathology			
OCD symptoms (OCI-R total)	23.32 (10.13)^2^	4.33 (5.76)	*U* = 107.00, *p* < 0.001
OCD washing (OCI-R washing)	4.49 (4.14)^2^	0.56 (1.22)	*U* = 347.00, *p* < 0.001
OCD obsessing (OCI-R obsessing)	7.45 (3.95)^2^	0.56 (0.92)	*U* = 168.50, *p* < 0.001
OCD ordering (OCI-R ordering)	3.77 (3.83)^2^	1.36 (1.92)	*U* = 695.00, *p* = 0.003
OCD checking (OCI-R checking)	5.36 (3.70)^2^	1.36 (2.19)	*U* = 347.50, *p* < 0.001
OCD neutralizing (OCI-R neutralizing)	2.25 (3.46)^2^	0.51 (1.10)	*U* = 781.50, *p* < 0.001
Obsessive beliefs (OBQ-44 total)	178.38 (58.15)^2^	103.62 (39.43)	*t*(90) = 7.17, *p* < 0.001
Responsibility/threat estimation (OBQ- RT)	67.30 (26.09)^2^	40.60 (16.61)*	*U* = 441.00, *p* < 0.001
Perfectionism/certainty (OBQ-PC)	69.11 (23.66)^2^	39.67 (16.36)	*t*(90) = 6.91, *p* < 0.001
Importance/control of thoughts (OBQ-ICT)	41.98 (18.67) ^2^	23.36 (11.27)	*U* = 438.00, *p* < 0.001
Depressive symptoms (PHQ-9)	13.38 (6.11)^2^	2.56 (2.23)	*U* = 88.00, *p* < 0.001
Non-acceptance of emotional responses (DERS)	18.47 (6.54)^3^	10.60 (4.85)	*U* = 221.50, *p* < .001
Anger—direct measure			
Trait anger (STAXI-2 trait subscale)	22.36 (7.35)	17.56 (4.22)	*U* = 623.50, *p* = 0.001
Anger suppression (STAXI-2 anger expression-in subscale)	18.23 (5.82)	14.87 (5.12)	*U* = 698.00, *p* = 0.005
Aggression (STAXI-2 anger expression-out subscale)	13.02 (4.67)	10.71 (2.26)	*U* = 762.00, *p* = 0.02
Anger control (STAXI-2 anger control subscale)	27.51 (6.05)	29.96 (6.80)	*U* = 913.00, *p* = 0.03
Aggressiveness—indirect measure			
Aggressiveness self-concept (*D*_*2*_-score Agg-IAT)	− 0.51 (0.37)^4^	− 0.54 (0.33)	*t*(91) = 0.52, *p* = 0.61
Error rates (Agg-IAT)	0.05 (0.03)	0.05 (0.05)	*U* = 1045.50, *p* = 0.79

*m* male, *f* female, *OCD* obsessive–compulsive disorder, *WST* test of word power, *SES-17* social desirability scale, *OCI-R* obsessive–compulsive inventory revised, *OBQ* obsessive-beliefs questionnaire- 44, *OBQ-RT* responsibility and threat estimation subscale, *OBQ-PC* perfectionism and intolerance of uncertainty subscale, *OBQ-ICT* importance and control of thoughts subscale, *PHQ-9* patient health questionnaire, *DERS* difficulty in emotion regulation questionnaire, *STAXI-2* State-Trait Anger Expression Inventory-2, *Agg-IAT* Aggressiveness Self-Concept Implicit Association Test

^1^based on *n* = 31

^2^based on *n* = 47

^3^based on *n* = 30

^4^based on *n* = 41

### Psychopathology

The Mini International Neuropsychiatric Interview (M.I.N.I.; [26]) is a structured diagnostic interview and was used for verifying the OCD diagnoses in patients as well as for excluding healthy controls if they fulfilled criteria of any mental disorder. The M.I.N.I. shows an excellent inter-rater reliability and a good to excellent test–retest reliability for both clinical and primary care populations [26]. For the present study, the M.I.N.I. was adapted according to Diagnostic-Statistical Manual-5 (DSM-5; [27]). The Yale-Brown Obsessive–Compulsive Scale (Y-BOCS; German version [28]), a semi-structured interview, was used to assess OCD symptom severity in patients. The Y-BOCS shows good internal consistency and inter-rater reliability [29]. The Y-BOCS entails ten items that can be scored on a five-point Likert scale ranging from 0 (no symptoms) to 4 (severe symptoms). A cutoff score of 16 has often been used for OCD [30]. The following measures were used as self-report questionnaires. The Obsessive–Compulsive Inventory-Revised (OCI-R; German version [31]) was used to assess distress caused by OCD symptoms on the subscales obsessive beliefs, washing, checking, neutralizing, ordering, and hoarding. The items are scored on a  five-point Likert-scale ranging from 0 (no symptoms) to 4 (severe). According to recommendations, the hoarding subscale was not included in the total score and was not analyzed in this study [32]. The total score ranges from 0 to 60 [33]. The OCI-R shows good reliability and validity scores [32]. The Patient Health Questionnaire-9 (PHQ-9; German version [34]) screens for a major depression episode according to the diagnostic criteria of the DSM-IV as well as depressive symptom severity [35]. The PHQ-9 shows good reliability and high validity [35].

### Aggressiveness

The State-Trait Anger Expression Inventory (STAXI-2; German version [36]) is a self-report measure consisting of 66 items on six subscales, which assess experience of anger (subscales: state anger, trait anger), suppressed anger or aggression (subscales: anger expression-in, anger expression-out), and control of anger (subscales: anger control-in, anger control-out). A four-point Likert-scale ranging from 1 to 4 is used. The STAXI-2 shows high internal consistency and high retest reliability for the subscales as well as high convergent and discriminant validity [36].

The Aggressiveness-Implicit Association Test (Agg-IAT [15]; adapted version by Cludius et al. [17]) was used as an indirect measure to assess the aggressiveness self-concept in all participants. The Agg-IAT is a reaction time task that reflects strength of associations between two different concepts (i.e., self vs. others, peaceful vs. aggressive). Participants are asked to sort words into certain categories by pressing one of two keys. Each key represents two categories. The Agg-IAT is based on the assumption that participants react faster when the two categories assigned to the same key have a stronger association in their memory network than the other combination. Stimuli from the target categories “self” and “others” and the attribute categories “peaceful” and “aggressive” were presented (stimuli are shown in Table 2). These stimuli were the same as in the previous study using the Agg-IAT in patients with OCD [17]. See Table 3 for an overview of the Agg-IAT procedure (i.e., block structure). Block order for the third and fifth block was counterbalanced. In each block, the category labels were presented and remained on the top left and top right corners of the screen. The stimulus was shown in the middle of the screen and had to be categorized. If participants pressed the wrong key, a red “X” appeared and participants had to press the other key. The stimulus disappeared once the correct key had been pressed. The following stimulus appeared 150 ms later. Response latencies were the time between the appearance of each stimulus and the correct response. The Agg-IAT was administered using the program Inquisit [37]. The Agg-IAT shows adequate psychometric properties. Its validity was supported by correlations with self-report measures and objective indicators of aggressive behavior in several studies [15, 16].

**Table 2:** Target and attribute stimuli used in the Agg-IAT

Target stimuli		Attribute stimuli	
Me	Others	Peaceful	Aggressive
Me (mir)	Miller (Müller)	Talk (reden)	Hunt (jagen)
My (mein)	Miller (Müllerin)	Conciliation (Versöhnung)	Revenge (Rache)
Me (mich)	Shoemaker (Schuster)	Conversation (Gespräch)	Punch (Faustschlag)
I (ich)	Shoemaker (Schusterin)	Exchange (Austausch)	Fight (Kampf)
Self (selbst)	Navigator (Lotse)	Compromise (Kompromiss)	Hit (Schlagen)
	Navigator (Lotsin)	Settlement (Einigung)	Avenge (rächen)
	Potter (Töpfer)	Agreement (Verständigung)	Retaliate (zurückschlagen)
	Potter (Töpferin)	Counseling (Beratung)	Threat (Drohung)
	Farmer (Bauer)	Agree (einigen)	Attack (Angriff)
	Farmer (Bäuerin)	Concede (nachgeben)	Beat (hauen)

The “others” stimuli were presented in both a female and a male version. The German version of the stimuli is listed in parentheses

**Table 3:** Overview of blocks presented in the Agg-IAT

Block number	Block name	Number of trails
1	Practice attribute categories: aggressive vs. peaceful	20 Trials
2	Practice target categories: me vs. others	20 Trials
3*	Test Incompatible: me + aggressive vs. others + peaceful	84 Trials
4	Practice attribute categories: peaceful vs. aggressive	20 Trials
5*	Test Compatible: me + peaceful vs. others + aggressive	84 Trials

Blocks 3 and 5 are the critical blocks (the first four trials were used as practice blocks and were thus discarded from the analyses)

### Mediators

To assess possible mediators, self-report questionnaires were employed. For all participants, the Obsessive-Beliefs Questionnaire-44 (OBQ-44; [18]) was used to assess OCD-related beliefs. The 44 items are scored on a seven-point Likert-scale ranging from 1 (disagree very much) to 7 (agree very much). Three subscales are formed: responsibility/threat estimation (16 items, range of total score from 16 to 120), perfectionism/certainty (16 items, range of total score from 16 to 120), and importance/control of thought (12 items, range of total score from 12 to 84). The OBQ-44 shows good internal consistency and good test–retest reliability [38]. The subscale responsibility/threat estimation (OBQ-RT) served as a mediator for the analyses. Social desirability was assessed via the SES-17 (“Soziale-Erwünschtheits-Skala-17”, social desirability scale; [39]). The scale consists of 17 items with dichotomous response format: 0 (I do not agree) and 1 (I agree). The SES-17 shows good test–retest reliability and acceptable construct validity. Non-acceptance of negative emotions was assessed via the respective subscale of the Difficulties in Emotion Regulation Scale (DERS; German version [40]). The subscale consists of six items which can be answered on a five-point Likert-scale ranging from 1 (almost never) to 5 (almost always). The total score of the subscale ranges from 6 to 30. The DERS shows good internal consistencies and test–retest reliability on all subscales and substantial correlations with other questionnaires on emotion regulation [40]. Verbal intelligence was assessed using the Test of Word Power (“Wortschatztest” (WST); [41]).

### Procedure

Assessments took place in single one-to-one settings. After giving their informed consent, participants completed—among other measures within the larger research project—a sociodemographic interview, the mental disorder screening interview (M.I.N.I.), the OCD symptom severity interview (Y-BOCS in patients only), the test of verbal intelligence (WST), and the battery of questionnaires on psychopathology (OCI-R; PHQ-9) as well as OCD-specific beliefs (OBQ-44), anger and aggression (STAXI-2), social desirability (SES), and non-acceptance of negative emotions (DERS). The Agg-IAT assessment was conducted using a laptop.

### Strategy of Data Analysis

Similar to previous studies using the Agg-IAT [15–17], error trials were discarded before computing a *D*_*2*_-score.

$$D2=\frac{\mathrm{Mean \ Response \ Latencies}\, \left(\mathrm{Incompatible \ Trials}\right)-\mathrm{Mean \ Response \ Latencies}\, \left(\mathrm{Compatible \ Trials}\right)}{\mathrm{Pooled \ Standard \ Deviation }\left(\mathrm{Incompatible \ and \ Compatible \ Trials}\right)}$$

Higher *D*_*2*_*-*scores indicate a faster reaction to me + aggressive/others + peaceful compared to me + peaceful/others + aggressive. Thus, higher *D*_*2*_-scores can be interpreted as a relatively increased aggressiveness self-concept. The reliability of the Agg-IAT was calculated following the procedure by Schmidt et al. [16]. The Agg-IAT revealed an acceptable to good reliability of Cronbach’s *α* = 0.74.

**Fig. 1:**
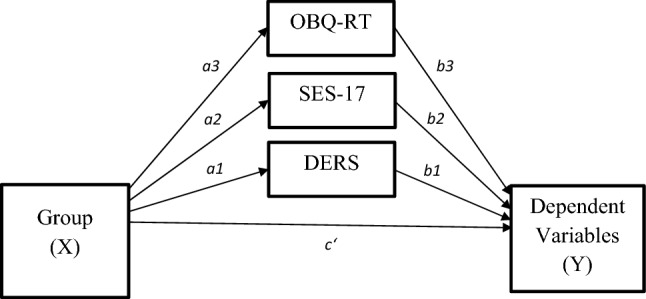
We computed three parallel mediation models with group (OCD, healthy) as predictor (X) and an inflated sense of responsibility (OBQ-RT subscale of the OBQ-44), non-acceptance of negative emotions (DERS), and social desirability (SES-17) as mediators to control for the inter-correlation between the three mediators. The dependent variables (Y) for these models were (1) aggressiveness self-concept (Agg-IAT), (2) trait anger (STAXI-2 trait subscale), and (3) anger suppression (STAXI-2 anger expression-in subscale). **a** = effect of independent variable on mediator, **b** = effect of mediator on outcome, **c′** = effect of group on outcome when controlling for the mediator

*t* tests (or, in case the data was not normally distributed, Mann–Whitney *U* tests) for independent samples were computed to test our hypotheses regarding group differences in (1) aggressiveness self-concept (Agg-IAT), (2) trait anger (STAXI-2 trait subscale), and (3) anger suppression (STAXI-2 anger expression-in subscale) between patients and healthy controls. We computed an analysis with a subsample of patients who could be classified as patients with checking-related symptoms of OCD according to the OCI-R (score of 6 or higher on the checking subscale; [31]). Three multiple mediation models (using trait anger, anger suppression, and aggressiveness self-concept as dependent variables) were computed using the SPSS-macro PROCESS (Version 3.3 [42]). For the mediation analyses, patients with OCD were coded with 1 and healthy controls were coded with 0. To correct for potential biases of non-normality in the sample, we bootstrapped results 10,000 times. See Fig. 1 for an overview of the parallel mediation model with the scores for anger/aggression as the dependent variable. Age and gender were added as covariates to the mediation models. Several exploratory *t* tests or Mann–Whitney *U* tests for independent samples were computed to test difference regarding the aggressiveness self-concept, trait anger, and anger suppression. In those analyses, the groups of patients were separated as follows: (1) patients with and without comorbid MDD, (2) patients with and without comorbid anxiety disorder, (3) patients with and without antidepressant medication (SSRIs), and then compared to the healthy control group. Effect sizes for *t* test results are expressed as Cohen’s *d*, whereby *d* ≈ 0.2 conventionally represents a small effect, *d* ≈ 0.5 a medium effect, and *d* ≈ 0.8 a large effect. An alpha level of 0.05 (two-tailed) was used for all statistical tests.

## Results

### Demographics and psychopathology

Patients and healthy controls did not differ on any of the demographic variables. As expected, patients with OCD scored significantly higher on all relevant psychopathological ratings, including OCD symptoms, dysfunctional beliefs (e.g., an inflated sense of responsibility), depressive symptoms, and non-acceptance of emotions (see Table 1). Patients with OCD reported a mean score of *M* = 25.15 (*SD* = 6.13) on the Y-BOCS total, which can be classified as severe symptoms of OCD [28], with *M* = 12.44 (*SD* = 3.54) on the obsessions subscale of the Y-BOCS, and *M* = 12.71 (*SD* = 3.28) on the compulsions subscale of the Y-BOCS. Patients with OCD reported an average of 12 years of disorder duration (*M* = 12.17, *SD* = 11.18). Most of the patients received an antidepressant medication (37.5%) or a combination of antidepressant and neuroleptic medications (27.1%). A minority of the patients received a neuroleptic agent (6.3%), a benzodiazepine (2.1%), or anticonvulsive agents (2.1%) only. The remaining patients were not medicated (25%). According to the results of the M.I.N.I., only a minority of the patients with OCD did not fulfill the criteria for a comorbid disorder (14.6%). The rest of the patients reported between 1 and 5 comorbid disorders with an average of *M* = 1.83 (*SD* = 1.24). Almost half of the patients with a comorbid disorder reported a comorbid anxiety disorder (45.8%) and/or an acute depressive disorder (54.2%).

### Group differences for anger and aggression

See Table 1 for an overview of anger and aggression scores. Data for the Agg-IAT (*D*_2_-scores), the OBQ total score, and the OBQ perfectionism and uncertainty subscales were normally distributed for both groups as assessed by the Shapiro–Wilk-Test, *p*s > 0.05. However, the remaining variables were not normally distributed for either or both of the groups (*p*s < 0.05). Mann–Whitney *U* tests were computed for the non-normally distributed data (see Table 1). As expected, patients with OCD scored higher on both trait anger (with a large effect, *d* = 0.78) and anger suppression with a medium effect (*d* = 0.64) as measured with the direct measure (STAXI-2). However, contrary to our hypotheses, patients with OCD did not differ from healthy controls in their aggressiveness self-concept as measured with the indirect measure (Agg-IAT; *d* = 0.09). The *t* tests comparing patients with checking-related symptoms of OCD according to the OCI-R (OCI-R checking subscale ≥ 6; *n* = 20) to healthy controls showed similar results (see online resource).^2^

### Mediation models

Intercorrelations between the predictor (group), the mediators (social desirability, non-acceptance of emotions, inflated sense of responsibility), and the dependent variables (aggressiveness self-concept, trait anger, anger suppression) are presented in Table 4. Results of the mediation models are presented in Table 5. The first parallel mediation model with the aggressiveness self-concept (Agg-IAT) as the independent variable showed no significant effect of group or any of the mediators (SES, DERS, OBQ-RT) on Agg-IAT scores at baseline. The second parallel mediation model indicated that group was indirectly related to trait anger (subscale of the STAXI-2) mediated by non-acceptance of emotions. Patients with OCD showed higher non-acceptance of negative emotions, whereas higher non-acceptance of emotions was related to higher trait anger scores. However, contrary to our expectations, an inflated sense of responsibility did not mediate this relationship. The third parallel mediation model similarly indicated that group was indirectly related to anger suppression (subscale of the STAXI-2). This was mediated by non-acceptance of emotions and an inflated sense of responsibility. Patients with OCD reported higher non-acceptance of emotions and an inflated sense of responsibility. Higher scores on those two scales predicted higher anger suppression. Both, the second and the third model, were full mediation models, in which 40% (trait anger) or 45% (anger suppression) of the variance was explained.

**Table 4:** Zero-order correlations between focal variables from the mediation analyses

Measure	1	2	3	4	5	6	7
1. Group (OCD, healthy)	–	− 0.52**	0.08	− 0.57**	− 0.05	− 0.37**	− 0.30**
2. Responsibility (OBQ-RT)		–	0.03	0.63**	− 0.08	0.36**	0.50**
3. Social desirability (SES-17)			–	− 0.04	− 0.13	− 0.22	− 0.20
4. Non-acceptance of emotions (DERS)				–	− 0.08	0.59**	0.61**
5. Aggressiveness self-concept (Agg-IAT)					–	− 0.13	− 0.05
6. Trait anger (STAXI-II trait anger)						–	0.46**
7. Anger suppression (STAXI-II anger-in)							–

*OBQ-RT* responsibility and threat estimation subscale of OBQ-44, *SES-17* Social Desirability Scale, *DERS* difficulty in emotion regulation questionnaire, *Agg-IAT* aggressiveness Implicit Association Test, *STAXI-2* State-Trait Anger Expression Inventory-2

**p < 0.01

**Table 5:** Mediation analyses with age and gender as covariates

Independent variable (IV)	Dependent variable (DV)	Mediator (M)	Effect of IV on M (a); *b* (SE), *t*, *p*	Effect of M on DV (b); *b* (SE), *t*, *p*	Indirect effect (ab); *b* [95% BC CI]	Direct effect (c′); *b* (SE), *t*, *p*	Total effect (c); *b* [95% BC CI]
In the whole sample (OCD, healthy)							
Group	Agg-IAT	OBQ-RT	**27.83 (5.05), 5.51, < 0.001**	− 0.00, (0.00), − 0.49, 0.62	− 0.03 [− 0.16; 0.09]	0.07 (0.10), 0.70, 0.48	− 0.05 [− 0.06; 0.07]
		SES	− 0.44 (0.49), − 0.91, .37	− 0.03 (0.02), − 1.44, 0.15	0.01 [− 0.02; 0.05]	0.07 (0.10), 0.70, 0.48	− 0.05 [− 0.06; 0.07]
		DERS	**7.93 (1.35), 5.86, < 0.001**	− 0.00, (0.01), − 0.58, 0.56	− 0.04 [− 0.16; 0.08]	0.07 (0.10), 0.70, 0.48	− 0.05 [− 0.06; 0.07]
Group	Trait anger	OBQ-RT	**27.83 (5.05), 5.51, < 0.001**	0.02 (0.03), 0.60, 0.55	0.56 [− 1.71; 2.77]	0.89 (1.62), 0.55, 0.58	**4.61 [1.77; 7.83]**
		SES	− 0.44 (0.49), − 0.91, 0.37	− 0.58 (0.30), − 1.88, 0.06	0.26 [− 0.35;1.02]	0.89 (1.62), 0.55, 0.58	**4.61 [1.77; 7.83]**
		DERS	**7.93 (1.35), 5.86, < 0.001**	**0.48 (0.12), 3.84, < 0.001**	**3.79 [1.41; 6.51]**	0.89 (1.62), 0.55, 0.58	**4.61 [1.77; 7.83]**
Group	Anger suppression	OBQ-RT	**27.83 (5.05), 5.51, < 0.001**	**0.06 (0.03), 2.18, 0.03**	**1.72 [0.20; 3.81]**	0.91 (1.38), 0.66, 0.51	**5.27 [3.22; 7.92]**
		SES	− 0.44 (0.49), − 0.91, 0.37	− 0.51 (0.26), − 1.94, 0.06	0.22 [− 0.24; 1.02]	0.91 (1.38), 0.66, 0.51	**5.27 [3.22; 7.92]**
		DERS	**7.93 (1.35), 5.86, < 0.001**	**0.42 (0.11), 3.95, < 0.001**	**3.32 [1.13; 5.81]**	− 0.91 (1.38), 0.66, 0.51	**5.27 [3.22; 7.92]**

*IV* independent variable, *DV* dependent variable, *M* mediator, *a* effect of independent variable on mediator, *b* effect of mediator on outcome, *BC CI* bootstrap-corrected confidence interval (based on 10,000 samples), *c* total effect of the independent variable on outcome (without the influence of the mediator in the model), *c*′ effect of group on outcome when controlling for the mediator, *OCD* obsessive–compulsive disorder, *Agg-IAT* aggressiveness implicit association test, *Trait anger* State-Trait Anger Expression Inventory-2 trait subscale, *A* *Anger suppression* State-Trait Anger Expression Inventory-2 anger-in subscale, *OCI-R* Obsessive–Compulsive Inventory Revised, *OBQ—RT* responsibility and threat estimation subscale of the Obsessive-Beliefs Questionnaire- 44, *Y-BOCS* Yale-Brown obsessive compulsive scale

Significant results are in bold

### Exploratory analyses for subgroups

The *t* tests or Mann–Whitney *U* tests were conducted separately for the subgroup of patients with (*n* = 19) and those without current comorbid MDD (*n* = 28) compared to healthy controls. Those with current comorbid MDD showed higher trait anger (*U* = 274.50, *p* = 0.02, *d* = 0.59) and anger suppression (*U* = 223.00, *p* = 0.003, *d* = 0.81) compared to healthy controls, those without current comorbid MDD only reported higher trait anger (*U* = 349.00, *p* < 0.001, *d* = 0.80) but not higher anger suppression (*U* = 475.00, *p* = 0.08, *d* = 0.59, but note the consistent effect size estimate). Neither of the groups differed from healthy controls regarding the aggressiveness self-concept as measured by the Agg-IAT (with comorbid MDD: *t*(63) = 0.43, *p* = 0.67, *d* = 0.11; without comorbid MDD: *t*(71) = 0.43, *p* = 0.67, *d* = 0.09). Similar results were revealed after dividing the subgroups into patients with (*n* = 26) and those without current anxiety disorder (*n* = 22). Patients with a comorbid anxiety disorder showed higher trait anger (*U* = 278.00, *p* < 0.001, *d* = 0.92) and anger suppression (*U* = 223.00, *p* < 0.001, *d* = 0.67), those without a comorbid anxiety disorder showed only higher trait anger (*U* = 345.50, *p* = 0.045, *d* = 0.50; anger suppression: *U* = 465.00, *p* = 0.69, *d* = 0.01) compared to healthy controls. No differences were found regarding the aggressiveness self-concept (with comorbid anxiety disorder: *t*(69) = 0.36, *p* = 0.72, *d* = 0.09; without comorbid anxiety disorder: *t*(65) = 1.42, *p* = 0.16, *d* = 0.37). Lastly, similar results were obtained for patients who were on antidepressants only (*n* = 18) and those who did not take any antidepressants (*n* = 17). Patients on antidepressants showed higher scores compared to healthy controls regarding trait anger (*U* = 219.00, *p* = 0.005, *d* = 0.76), anger suppression (*U* = 226.00, *p* = 0.006, *d* = 0.73), but no difference regarding the aggressiveness self-concept (*t*(61) = 1.29, *p* = 0.20, *d* = 0.36). Patients who did not take antidepressants reported higher trait anger (*U* = 165.50, *p* = 0.001 *d* = 0.97) than healthy controls but did not differ regarding anger suppression (*U* = 280.00, *p* = 0.19, *d* = 0.42) or an aggressiveness self-concept (*t*(60) = 1.25, *p* = 0.22, *d* = 0.35).

## Discussion

This study was the first to incorporate both direct and indirect measures into the assessment of anger and aggressiveness self-concepts in patients with OCD. Furthermore, it assessed possible mediators that could explain the relationship between OCD and anger or anger suppression, namely an inflated sense of responsibility, non-acceptance of emotions, and social desirability. Based on psychodynamic [43] and cognitive theories [3], as well as previous studies (e.g., [8, 11, 17]), we assumed differences between patients with OCD and healthy controls regarding trait anger and anger suppression. Furthermore, we expected that an inflated sense of responsibility would mediate the relationship between OCD and anger or anger suppression.

### Trait anger and suppressed anger according to a direct measure

As expected, patients with OCD reported higher trait anger and anger suppression in the direct self-report (STAXI-2) compared to healthy controls. Both results are in line with the majority of previous studies [8–11]. Higher self-reported anger suppression supports the assumptions of Rachman [3] and Freud [2], who suggested that anger is suppressed rather than expressed in OCD. According to Rachman [3], individuals with OCD would experience feelings of guilt and a tendency to blame themselves rather than others, because they feel responsible for preventing harm and failing to fully do so.

### Aggressiveness self-concept as measured by an indirect measure (IAT)

Contrary to our expectations, we found no difference in the aggressiveness self-concept as measured by an indirect measure (Agg-IAT) between patients with OCD and healthy controls. The exploratory analysis that compared patients with checking-related compulsions (according to the OCI-R) and healthy controls revealed similar results. In the first study that used an Agg-IAT in patients with OCD [17], the whole sample of patients with OCD and healthy controls did not differ, however a subsample of patients with checking-related compulsions (according to the OCI-R) showed a more peaceful self-concept compared to the control group. Even though scores of patients with OCD and patients with checking-related symptoms of OCD did not statistically differ in the previous study, they showed a nominal difference (checking: *D*_*2*_-score = − 0.67; whole OCD sample: *D*_*2*_*-*score = − 0.54). Yet, the *D*_*2*_-scores in this study were very similar between patients with checking-related compulsions and the whole OCD sample (checking: *D*_*2*_-score = − 0.52; whole OCD sample: *D*_*2*_-score = − 0.51). Several reasons could explain the difference between the aggressiveness self-concept of patients with checking-related symptoms in the previous study and this study. One reason could be that patients in the previous study had more severe symptoms of OCD, especially checking-related symptoms. The patients with checking-related symptoms in this study had a descriptively slightly higher Y-BOCS score (Cludius et al. 2017, *M* = 24.81; this study, *M* = 27.10, *t*(45) = 1.93, *p* = 0.06). However, the disorder duration was descriptively slightly longer in the previous study (Cludius et al. 2017, *M* = 16.94; this study, *M* = 11.55, *t*(45) = 1.77, *p* = 0.08). The score for checking-related symptoms on the OCI-R was similar (Cludius et al. 2017, *M* = 9.14; this study, *M* = 9.00, *t*(45) = 0.24, *p* = 0.81). Therefore, differences in disorder severity do not seem likely to explain differences in the aggressiveness self-concept in this study compared to the previous study. Another reason could be that the sample in this study was too small to replicate the results of the previous study. Pooling the Agg-IAT effect sizes from the present and the former studies [17] in a mini meta-analysis (fixed effects; Hedges *g*; 95% CI) also resulted in non-significant group differences [healthy controls vs. all patients *g* = 0.05 (− 0.25; 0.36); healthy controls vs. patients with checking-related compulsions *g* = 0.28 (− 0.10; 0.66)]. Taken together, it could be possible that the more peaceful self-concept in the previous study was a mere false positive finding and that the modest sample sizes in both studies render replications of previous results difficult. Another explanation could be that the Agg-IAT may not be specific enough to assess implicit aggressiveness in OCD. The stimulus words used in the Agg-IAT refer to overt aggression (e.g., fight, revenge) which may not appropriately depict anger and aggressiveness related to OCD. Future studies could establish and use indirect measures which tap more specifically into the concept of suppressed anger.

### Possible reasons for elevated anger and anger suppression scores in OCD

First, social desirability, non-acceptance of negative emotions, and an inflated sense of responsibility were tested as possible mediators between OCD and anger or anger suppression. Social desirability did not mediate the relationship between group and anger or anger suppression scores.Patients and healthy controls did not differ on the social desirability scale (SES-17). Thus, it is very unlikely that patients report anger differently because of differences in how they present themselves, for example, due to high moral standards.

Second, an inflated sense of responsibility did not mediate the relationship between group and anger, but mediated the relationship between group and anger suppression. This is in line with the theory by Rachman [3], who assumed that anger would be suppressed rather than expressed in patients with OCD, because they take full responsibility. According to this, anger suppression would be a consequence of OCD. However, as this study did not measure temporal relationships, the causality remains to be tested.

Third, non-acceptance of negative emotions mediated the relationship between group and trait anger as well as anger suppression. To the best of our knowledge, no previous study has assessed that link in OCD. Regarding depression, a study showed that emotional suppression was only associated with symptoms of depression when moderated by non-acceptance of emotions [44]. In an ecological momentary assessment study in patients with borderline-personality disorder, non-acceptance of negative emotions increased the number of negative complex emotions prior to symptoms of self-injury [45]. This may indicate that lower acceptance of negative emotions could lead to an increase in anger, which leads to a greater number of OCD symptoms. However, laboratory or ecological momentary assessment studies are needed to assess this causal link. Furthermore, next to OCD, MDD, and borderline personality disorder, non-acceptance of negative emotions has been found to be associated with other mental disorders, such as eating disorders [46] and social anxiety disorder [47]. This might indicates a transdiagnostic factor which likely refers to all kinds of internalizing disorders [48]. Future research is necessary to assess whether the link between non-acceptance of negative emotions and anger or anger suppression is specific to OCD or whether it is associated with other (groups) of disorders.

Fourth, our analyses regarding the subgroups of patients (with or without a current comorbid MDD or current comorbid anxiety disorder, intake or no intake of antidepressants) give an indication that group differences to healthy controls, regarding trait anger, are not attributable to comorbid MDD, anxiety disorder or to the intake of antidepressant medication. However, it is possible that comorbidity explains some of the difference regarding anger suppression. Only patients with current comorbid MDD reported higher anger suppression compared to healthy controls, whereas patients with no current comorbid depression did not show significantly higher anger suppression scores. Similar results were found when dividing the group of patients into those with and without anxiety disorders and those who use antidepressants and those who do not. Notably, the sample sizes of the subgroups were quite small and the non-significant effects showed, in some cases, moderate effect size estimates. Nevertheless, this study cannot fully differentiate whether higher anger suppression in OCD is specifically attributable to OCD or rather to MDD, anxiety disorders, or the intake of antidepressants.

### Limitations

This study shows a number of limitations. First, most patients with OCD reported a comorbid disorder according to the M.I.N.I., mostly anxiety disorders or MDD. Furthermore, patients on average showed moderate depressive symptoms on the PHQ-9. Some studies indicated that anger and anger suppression could be moderated by depressive symptoms, as relationships between OCD and anger were no longer significant after controlling for depression [11, 13]. However, by controlling for depressive symptoms it is likely that relevant variance in the relationship between OCD and anger is removed (see [49]). Also, many patients were medicated, mostly with antidepressants (SSRIs). The serotonergic system has been linked to aggression, whereas antidepressants have been found to reduce aggression in patients with a personality disorder [23]. However, excluding patients without comorbid disorders or antidepressant medication would have reduced the ecological validity of our study as comorbidity rates, especially with MDD and anxiety disorders, are generally high in OCD [50]. Future studies should be designed to test those possible mediators in the relationship between OCD and anger or anger suppression, for example, by including clinical control groups or recruiting larger samples. Second, an inflated sense of responsibility was measured in one dimension with overestimation of threat, as those load on the same factor [18]. Our results related to the inflated sense of responsibility are limited insofar, as we cannot draw conclusions about each single dysfunctional belief but only the respective bias dimension. Third, as stated above, this study gives an indication as to whether certain mediators are predictive of anger or anger suppression. However, it does not prove causal links. Future studies should use laboratory or longitudinal designs to test if an inflated sense of responsibility and non-acceptance of emotions are risk factors for the development of anger or anger suppression.

### Clinical implications

Cognitive behavioral therapy with exposure and response prevention (ERP) is the gold standard intervention in OCD. However, about 30% of those who undergo ERP do not profit sufficiently. Additionally, about a quarter of patients with OCD refuse to engage in ERP at all, and 30% discontinue the treatment prematurely [51]. Our results may give some indication that training adaptive emotion regulation strategies may be beneficial as an add-on to ERP, which could also reduce drop-out rates and enhance remission. First, our preliminary evidence shows that targeting anger suppression may be important. According to Rachman [3], anger can arise in patients with OCD due to an inflated sense of responsibility, when additional demands are made on the individual. Thus, the person may feel overwhelmed by tasks that he/she should fulfill in therapy, especially when engaging in ERP. As a consequence, anger may be provoked during therapy and may even be directed towards the therapist. It might be helpful for patients to know that it has not been empirically found that patients show a more aggressive self-concept, neither in this study nor in a previous study [17]. Furthermore, it could be beneficial if patients learned to reduce anger suppression, for example, using more adaptive emotion regulation strategies. Similarly, as non-acceptance of emotions mediated the relationship between OCD and anger or anger suppression, patients could also profit from a training to enhance emotional acceptance. For example, emotion regulation therapy [52] or acceptance and commitment therapy approaches [53] could help patients with OCD learn to identify and accept instead of avoid their emotions and to use the emotional information to identify needs or guide behavior and thinking rather than suppressing emotions. In a single-case study, an emotional awareness training was combined with exposure to OCD-specific and non-specific emotional cues [54]. This study provided first evidence that learning acceptance of emotions could improve exposure therapy, at least for some patients. Acceptance and commitment therapy, which aims to teach patients to accept negative emotional states, has shown some efficacy in OCD [55] but might not increase efficacy when added to ERP [56]. However, as the acceptance and commitment approach is not inferior [53], it might be an alternative in cases, in which the standard approach does not work (or is not feasible). Third, meta-cognitive interventions, such as meta-cognitive training [24], could help to reduce dysfunctional beliefs, such as an inflated sense of responsibility, which may lead to a further reduction of anger and anger suppression.

## Conclusion

This is the first study that combines an assessment by a self-report measure of anger and aggression and an indirect measure of an aggressiveness self-concept in patients with OCD. As expected, patients with OCD reported higher anger and anger suppression scores compared to healthy controls in the direct measures. Contrary to our hypothesis, patients with OCD did not differ from healthy controls regarding the aggressiveness self-concept as measured with an Implicit Association Test. Furthermore, several mediators in the relationship between OCD, anger, and anger suppression were tested. Non-acceptance of emotions served as a mediator between OCD and anger as well as OCD and anger suppression. An inflated sense of responsibility mediated the relationship between OCD and anger suppression. These results suggest that anger and anger suppression may be closely related to OCD. However, future studies are necessary to test whether anger suppression is specifically linked to OCD or whether higher anger suppression in OCD is due to comorbidities of medication intake. Furthermore, whether or not anger or anger suppression are important to the development or maintenance of the disorder should be tested in experimental laboratory or longitudinal studies. Our results suggest that maladaptive emotion regulation strategies (e.g., non-acceptance of emotions) as well as dysfunctional beliefs (e.g., an inflated sense of responsibility) may lead to higher anger and aggression and should be targeted in clinical interventions.

## Electronic supplementary material

Below is the link to the electronic supplementary material.

Supplementary file1 (DOCX 17 kb)

## Footnotes

1 Anger is usually defined as an affective state with feelings such as annoyance or fury, whereas aggression is defined as destructive behavior directed towards others. However, both concepts show substantial overlap and aggression is motivated by anger [57].

2 As age and education differed between the groups on a trend level, we also computed the analyses including age and education as covariates. However, results did not change for either the comparison of trait-anger, anger-suppression, or aggressiveness self-concept (Agg-IAT).
