# Supplementary material for: Anger and aggressiveness in obsessive–compulsive disorder (OCD) and the mediating role of responsibility, non-acceptance of emotions, and social desirability
Source: Eur Arch Psychiatry Clin Neurosci. 2020 Nov 5;271(6):1179–91. doi: 10.1007/s00406-020-01199-8 (PMC8354876; doi:10.1007/s00406-020-01199-8)
Supplement: Supplementary file 1 — Supplementary file1 (DOCX 17 kb) [file 406_2020_1199_MOESM1_ESM.docx]

Supplement. Demographic, Psychopathological, and Anger Data: Mean (Standard Deviation) of patients with checking-related symptoms of OCD compared to healthy controls

|  | Patients with  checking-related  symptoms of OCD  (*n* = 20) | Healthy controls  (*n* = 45) | Statistics |
| --- | --- | --- | --- |
| *Demographic characteristics* |  |  |  |
| Age | 35.35 (10.09) | 37.29 (14.28) | U = 518.00, p = .42 |
| Education (years) | 11.25 (1.48) | 11.93 (1.57) | U = 480.50, p = .18 |
| Sex (m/f) | 13/7 | 20/25 | χ^2^(1) = 2.34, *p* = .13 |
| Verbal intelligence (WST) | 101.25 (13.49) | 106.24 (8.88) | U = 473.00, p = .18 |
| Social Desirability (SES-17) | 9.56 (1.67)^1^ | 9.24 (1.96) | U = 424.00, p = .96 |
| *Psychopathology* |  |  |  |
| Y-BOCS total | 27.10 (4.17) | - | *-* |
| Y-BOCS obsessions | 13.50 (2.35) | - | *-* |
| Y-BOCS compulsions | 13.60 (2.37) | - | *-* |
| Illness duration (years) | 11.55 (2.27) | - | *-* |
| OCD Symptoms (OCI-R total) | 30.30 (9.06) | 4.33 (5.76) | U = 27.00, *p* < .001 |
| OCD Washing (OCI-R washing) | 4.90 (3.81) | 0.56 (1.22) | U = 173.00, *p* < .001 |
| OCD Obsessing (OCI-R obsessing) | 7.55 (3.75) | 0.56 (0.92) | U = 95.00, *p* < .001 |
| OCD Ordering (OCI-R ordering) | 4.95 (3.95) | 1.36 (1.92) | U = 313.50, *p* = .001 |
| OCD Checking (OCI-R checking) | 9.00 (2.13) | 1.36 (2.19) | U = 39.50, *p* < .001 |
| OCD Neutralizing (OCI-R neutralizing) | 3.90 (4.13) | 0.51 (1.10) | U = 323.50, *p* < .001 |
| Obsessive Beliefs (OBQ total) | 198.85 (55.88) | 103.62 (39.43) | U = 87.50, *p* < .001 |
| Responsibility/Threat Estimation (OBQ- RT) | 78.50 (23.69) | 40.60 (16.61)* | U = 102.00, *p* < .001 |
| Perfectionism/Certainty (OBQ-PC) | 77.45 (20.81) | 39.67 (16.36) | *t*(63) = 7.89, *p* < .001 |
| Importance/Control of Thoughts (OBQ-ICT) | 41.90 (19.41) | 23.36 (11.27) | U = 213.00, *p* < .001 |
| Depressive Symptoms (PHQ-9) | 13.65 (7.46) | 2.56 (2.23) | U = 49.50, *p* < .001 |
| Non-acceptance of emotional responses (DERS Nonaccept) | 17.93 (6.37)^2^ | 10.60 (4.85) | U = 131.00, *p* < .001 |
| *Anger - Direct Measure* |  |  |  |
| Trait Anger (STAXI-2 trait subscale) | 22.60 (6.98) | 17.56 (4.22) | U = 360.00, *p* = .007 |
| Anger Suppression (STAXI-2 anger expression-in subscale) | 18.85 (6.61) | 14.87 (5.12) | U = 372.00, *p* = .01 |
| Aggression (STAXI-2 anger expression-out subscale) | 12.25 (4.18) | 10.71 (2.26) | U = 486.00, *p* = .23 |
| Anger Control (STAXI-2 anger control subscale) | 27.40 (5.99) | 29.96 (6.80) | U = 436.50, *p* = .08 |
| *Anger - Indirect Measure* |  |  |  |
| Aggressive self-concept (*D_2_*-score Agg-IAT) | -0.52 (0.34) | -0.54 (0.33) | *t*(63) = 0.27, *p* = .79 |
| Error rates (Agg-IAT) | .04 (.03) | .05 (.05) | U = 580.50, *p* = .96 |

*Note*: m = male, f = female, OCD = obsessive-compulsive disorder, WST = Test of Word Power, OCI-R = Obsessive-Compulsive Inventory Revised, OBQ = Obsessive-Beliefs Questionnaire- 44, OBQ-RT = Responsibility and threat estimation subscale, OBQ-PC = Perfectionism and intolerance of uncertainty subscale, OBQ-ICT = Importance and control of thoughts subscale, PHQ-9 = Patient Health Questionnaire, DERS = Difficulty in Emotion Regulation Questionnaire, STAXI-2 = State-Trait Anger Expression Inventory-2, Agg-IAT = Aggressiveness Implicit Association Test.

^1^ based on *n* = 16

^2^ based on *n* = 15

Anger and Aggressiveness in Obsessive-Compulsive Disorder (OCD) and the Mediating Role of Responsibility, Non-acceptance of Emotions, and Social Desirability, Journal: [European Archives of Psychiatry and Clinical Neuroscience](https://www.springer.com/journal/406/)), authors: Barbara Cludius, Anna K. Mannsfeld, Alexander F. Schmidt, and Lena Jelinek, corresponding authors: Barbara Cludius, Department of Psychiatry and Psychotherapy, University Medical Center Hamburg-Eppendorf, Hamburg, Germany, barbara.cludius@psy.lmu.de
